# Circulating Melanoma Cells: Scoping the Target

**DOI:** 10.3389/fonc.2013.00189

**Published:** 2013-08-12

**Authors:** Powrnima Joshi, Maciej Zborowski, Pierre L. Triozzi

**Affiliations:** ^1^Department of Biomedical Engineering, Lerner Research Institute, Cleveland ClinicCleveland, OH, USA; ^2^Taussig Cancer Institute, Cleveland ClinicCleveland, OH, USA

## Circulating Melanoma Cells

Molecular markers have been increasingly applied in cancer to assess metastatic risk and to guide treatment, including in melanoma, where assessment of BRAF mutations in tumor tissues to determine suitability for treatment with vemurafenib is now routine (1). Static assessment of tumor tissues, however, does not indicate whether tumor cells are being shed or whether treatment is reducing metastasis. Because melanoma metastasizes hematogenously, examination of circulating melanoma cells (CMC) is a logical as well as a convenient alternative to the examination of tumor tissues. A reliable assessment of CMC numbers and molecular signatures could have major clinical impact. The failure to demonstrate a survival advantage for adjuvant treatment might be linked to inadequate disease staging and, consequently, inadequate assessment of relapse risk. Because CMC may indicate systemic subclinical disease, their detection and analysis may be useful not only for staging/prognosis but also for assessing response to adjuvant therapy. The discovery of CMC theoretically would also allow for earlier detection of metastasis. This could potentially increase the effectiveness of existing therapies. Serial CMC assessments during treatment may allow for the earlier assessment of response, sparing non-responding patients toxicities. Serial CMC assessments could help determine the mechanisms of resistance and suggest interventions to address them. Furthermore, not all patients with melanoma are candidates for surgery to obtain tissue for analysis of molecular markers, and CMC would provide a liquid biopsy of sum total of tumors at all the sites in the patient.

## Polymerase Chain Reaction Approaches

Polymerase chain reaction (PCR)-based techniques that detect the expression of the mRNA/transcripts of melanocyte-associated factors, such as tyrosinase and Melan-A, in nucleated blood cells and present in cell free fraction have been best studied clinically. They have demonstrated promise in melanoma surveillance and in monitoring adjuvant and metastatic therapy (2–4). They can also be combined with assessment of circulating DNA for melanoma-associated mutations, which in itself can be used to infer the presence of CMC (5). No PCR-based approach, however, has been validated for clinical use, due to limitations in consistency and high false negative rates. These approaches cannot quantify the number of CMC, and morphologic evaluation of the cells cannot be obtained. The presence of normal cellular transcripts by leukocytes, which contribute most of the total nucleotides extracted, may dilute those that are tumor-related, even following substantial enrichment for CMC (6). Furthermore, tumor heterogeneity may lead to clones of cells that do not express the melanocyte marker. There are technical issues. RNA is inherently labile (7). Differences in the PCR methodologies applied as well as differences in data interpretation may also be responsible for the disparate findings of various studies. Because cells are not captured, the ability to evaluate changes in targets or biological characteristics is limited, particularly in the context of the tumor heterogeneity that characterizes melanoma (8).

## Cytometric Approaches

Techniques that isolate and enumerate morphologically identified CMC have also been studied. Although several steps can be involved, molecular characterization of the CMC isolated has been accomplished. Typically, cytometric approaches have two components: a preparative and an analytical one. Preparative enrichment is required because CMC are rare in the blood, at counts lower than 10/ml of whole blood (as low as 1 CMC per 1,000,000 leukocytes). The goal is to increase sensitivity. The analytical step eliminates the non-relevant blood cells in the enriched fraction. The goal is to increase specificity. Here the greatest concern is again false negative results. Despite an abundance of potential markers, a consensus on how melanoma cells circulate, their phenotype, and the optimal capture reagent have not been established. Tumor cells in circulation may not always exhibit the criteria used to identify them in the context of tissue biopsy. The CellSearch^®^ system (Veridex LLC, Raritan, NJ, USA), a cytometric approach based on the immunomagnetic capture of circulating EpCAM-positive tumor cells, has been approved by the U.S. Food and Drug Administration to monitor the effectiveness of therapy in patients with metastatic breast, colorectal, and prostate carcinomas (9). No cytometric method has been validated for clinical use in melanoma. Representative approaches applied include the following:

### Physical

Density gradient separations with for example, Ficoll, combined with elimination of erythrocytes using isotonic ammonium chloride lysis method have been applied (10). The advantages of these approaches are simplicity and lower costs. However, these techniques typically have unacceptably high cell losses and thus lack sensitivity. Several platforms, including filter-based micro-devices, or microfluidic devices, using size as the capture method have been described (11, 12). Given their heterogeneity, it is not clear that large size is a sufficient criterion to capture all CMC. Dielectrophoretic forces have been applied, and cells of different types have been separated, without interfering with their viability, according to their dielectric and hydrodynamic flow properties (13). These approaches may be applicable to CMC but have not yet been effectively applied clinically.

### Immunomagnetic – positive selection

Circulating melanoma cells have been positively selected using immunomagnetic cell enrichment technique with antibody to, for example, the high molecular weight-melanoma-associated antigen (HMW-MAA). Cells isolated have been assessed for BRAF status (14). CellSearch^®^ technology has also been applied to CMC (15). Magnetic particles are tagged with antibodies against the melanoma-associated cell-surface antigen CD146 and captured in a magnet. These cells are detected using microscopy by fluorescently tagged antibody to HMWMAA (15). The caveat of this technology is the non-specificity of the capture antigen. CD146 is also expressed on circulating endothelial cells. It is also not known whether all CMC express CD146. Hence, sensitivity of this approach is unclear. Multiple antibody-bound beads were suggested to increase the sensitivity of positive selection of CMCs, however, the approach may add to the complexity of the microscopic image analysis and interpretation (16).

### Immunomagnetic – negative selection

Negative immunomagnetic selection is an attractive approach for isolating CMC in the absence of reliable CMC surface markers. Antibodies tagged with magnetic particles against CD45 antigen present ubiquitously on leukocytes are used to magnetically deplete a blood sample of white cells. These remaining non-magnetic cells are analyzed for CMC with melanocyte-associated markers, such as Melan-A/MART-1, HMB-45, and S100B. The advantage of this technique is unbiased capture of non-leukocytic cells, and disadvantage is lower purity of the CMC due to less than 100% capture of leukocytes. Negative separation has been successfully used to isolate CMC, and also offers the possibility of molecular characterization (17, 18). Although CMC prepared by this method are not pure, due to less than complete depletion of leukocytes, this disadvantage may be outweighed by the presumed complete capture of all types of CMC.

### Automated cytometric methods

Flow cytometry methods using antibodies against melanocyte determinant have been used to identify and capture CMC. However, the throughput is low, and the rarity of CMC make this technique by itself less practical (19). High speed scanning microscopy techniques such as fiber-optic based automated scanning technology and laser microdissection may be used in identifying and enumerating CMC that are identified with fluorescently tagged melanocyte-associated markers (20, 21). Combining these technologies with unique chip-based substrates has made possible the molecular characterization of single melanoma cells (21).

## Summary

Polymerase chain reaction-based approaches to enumerate rare and heterogeneous CMC have demonstrated promise but do not allow for morphologic or molecular analysis of specific cell populations. Although cytometric approaches are in clinical use in the management of patients with carcinomas, the development of similarly approved technology for CMC has proven challenging because of the lack of specific, cell-surface, CMC capture antigen(s). Analysis of the captured cells, such as the identification of molecular targets or special biological characteristics, with current methods can also be cumbersome. Thus, there remains a need for the development of a reliable, efficient platform to isolate, enrich, and characterize CMC in blood. Molecular assessments are now impacting melanoma management. Given the multitude of therapeutic targets emerging, whole genome sequencing adapted to enriched CMC obtained from peripheral blood samples will be necessary for meaningful evaluation of therapeutic directions, and given tumor heterogeneity, it will most likely need to be aimed at the single cell level.
